# Awareness and Practice of Najran University Students Towards Common Problems Related to Ear, Nose, and Throat

**DOI:** 10.7759/cureus.51178

**Published:** 2023-12-27

**Authors:** Abdullah M Assiri, Asaiel AL Hadi, Saleh Y Al Hareth, Meshal Alwabel, Enas M Al Hadi, Khalid M Alkhalifah

**Affiliations:** 1 Surgery, College of Medicine, Najran University, Najran, SAU; 2 College of Medicine, Najran University, Najran, SAU; 3 Unaizah College of Medicine and Medical Sciences, Qassim University, Unaizah, SAU

**Keywords:** university student, knowledge, public health and safety, orl-hns, ent

## Abstract

Objective: The objective of this study was to evaluate the awareness and practice of Najran University students toward common problems related to the ear, nose, and throat.

Methods: A cross-sectional study was utilized in this research, employing data from a sample of 429 students at Najran University. The participants completed a self-administered questionnaire and ensured anonymity. The questionnaire used in this study had been previously validated.

Results: The sample for the current study primarily consisted of students aged more than 20 years (84.1%; n = 361), with a predominance of females (69.0%; n = 296). The majority of them were in health colleges (45.2%; n=194). The study results show that 37.8% (n = 162) had a good knowledge level, while 62.2% (n = 267) had poor knowledge about problems related to ENT. The vast majority, 87.2% (n = 374), believed that going to the hospital was the appropriate action to take in cases of acute ENT problems. The results established a statistically significant association between age, gender, health college, college, and department with p-values <0.005 (0.002*, 0.001*, 0.003*, and 0.005*), respectively, and the level of knowledge about problems related to ENT. There was no statistically significant association between nationality, clinical history of the participants, and the level of knowledge about problems related to ENT (p > 0.005).

Conclusion: The study revealed that 37.8% of the participants had good knowledge about problems related to the ears, nose, and throat. The participants older than 20 years had better knowledge of common ENT problems than those younger than 20 years. Female participants showed a higher level of knowledge and awareness of problems related to the ears, nose, and throat. The study noted that the participants in Health College, the faculty of medicine, and the third academic level had good knowledge about problems related to ENT. The study established that going to the hospital was the appropriate action to be taken in case of a sudden ENT problem. Therefore, we recommend concerted efforts be made among the medical community to increase knowledge about common ENT problems.

## Introduction

Otorhinolaryngology (ORL), also known as ear, nose, and throat (ENT) medicine, is a specialized branch of medicine focused on the diagnosis and treatment of disorders related to the ear, nose, and throat, as well as the head and neck. The importance of ORL knowledge and awareness among the general population cannot be overstated, as many common ENT conditions can significantly impact an individual's quality of life and may lead to more severe complications if not addressed on time [1,2]. However, diseases of the ENT are of extreme importance due to the morbidities that can result in physiological function impairment. The aforementioned difficulties encompass impairments in flavor, olfaction, speech, respiration, swallowing, phonation, protection of the lower respiratory tract, hearing, and secretion clearance [3,4].

Among the leading reasons individuals consult primary health care physicians around the world are symptoms associated with ENT [5,6]. Based on the WHO, approximately 278 million people globally suffer from bilateral hearing loss ranging from moderate to severe [7]. It was reported that 16.1% of adults in the United States (US) had encountered hearing loss during the period from 2003 to 2004 [8]. In 2004, an estimation placed the prevalence of rhinosinusitis among nasal diseases among the adult population of the United States at 16% [9].

In Saudi Arabia, there is a growing concern about the level of knowledge and awareness of ORL-related issues among the younger population, specifically university students. This concern arises from the increasing prevalence of ENT problems in the country, with sinusitis, otitis media, and tonsillitis frequently reported among the population [10]. Early recognition and appropriate management of these conditions are essential to prevent complications and improve the overall health of affected individuals [11]. The reports of local knowledge and attitudes about ENT-related issues were insufficient. In a previous study, it was determined that 2.3% of individuals residing in the city of Riyadh showed exceptional knowledge, while 18.4% demonstrated a high level of knowledge. The majority of individuals (79.4%) have limited knowledge of ENT-related issues [12]. Another study was among school and university students in Makkah City, which showed a significant proportion of participants (42.22%) showed a moderate comprehension of ENT issues, while 41.48% showed a low level of understanding. On the other hand, only 16.30% of the respondents displayed a good degree of knowledge in this domain [10].

Najran City, located in the south of Saudi Arabia, has a substantial number of university students who may be at risk for ENT-related issues due to various factors, such as environmental conditions, lifestyle habits, and limited access to specialized healthcare services in the region [13]. However, to date, there is limited information available about the knowledge and awareness of common ORL-related issues among Nagran or university students.

To address this knowledge gap, this cross-sectional study aims to assess the level of knowledge and awareness of common ORL-related issues among university students in Najran City, Saudi Arabia. This study's findings will help identify gaps in ORL knowledge and awareness, inform the development of targeted educational interventions, and contribute to the improvement of ENT health outcomes in the region.

## Materials and methods

Study design and population

This study employs a cross-sectional design to evaluate the knowledge and awareness of common otorhinolaryngology-related issues among students at Najran University in Saudi Arabia. The target population consists of university students from different faculties and academic levels.

Sample size and sampling technique

After determining the required sample size using a standard formula for cross-sectional studies, it was 376. And we employ a convenience sampling technique to select participants based on their accessibility and willingness to participate from various faculties and academic levels at Najran University. Although this method is easy to implement, it may result in a biased sample, limiting the generalizability of the findings.

Data collection tool

The study uses a structured, self-administered questionnaire to collect data from the participants. After permission, we used the questionnaire used by Jaladdin et al. (2023) [10]. The questionnaire was divided into four sections. The first section was to explain the goal of our study and the consent of the participant. The section was for demographic information about the participants. The third section was for the clinical history, with four questions. The fourth section contains a series of multiple-choice questions with three answer options, except for the last two: "The appropriate action in case of hearing loss" had four answers and "Usually get information and knowledge about ENT issues" had six answers. The questionnaire was developed by board-certified otorhiolarngiologists. However, before conducting this survey, we performed content validity testing to ensure that the questions covered all aspects of the intended construct or topic. After validity, a reliability test was done using Cronbach’s alpha. The result indicated very good internal consistency and reliably measured the practice and level of knowledge of ear, nose, and throat-related problems.

Data analysis

After collecting the questionnaires, researchers enter the data into a statistical software program for analysis. They use descriptive statistics, such as frequencies, percentages, means, and standard deviations, to summarize the demographic information and responses related to knowledge and awareness of otorhinolaryngology issues. They also employ inferential statistics, including chi-square tests and t-tests, to examine associations between demographic variables and awareness levels.

Ethical considerations

The study adheres to ethical considerations, such as obtaining ethical approval from the Committee of Scientific Research and Conferences at Najran University, which issued approval No. 4\3\2023, obtaining informed consent from participants, ensuring confidentiality and anonymity, and respecting participants' rights to withdraw from the study at any time.

## Results

Table 1 shows that a total of 429 participants completed the questionnaire. The vast majority (84.1%, (n=361) of participants were more than 20 years old, with more than half (69.0%, (n=296) being female. 45.2% (n=194) of the students who participated in the survey studied at Health College, with 22.1% (n=95) in the faculty of medicine and 8.6% (n=37) in the faculty of nursing. The majority (16.8%; (n=72) of the participants were in the third academic level, with the vast majority (98.8%; (n=424) being Saudi nationals.

**Table 1:** Demographic information of the participants (N=429) Demographic information is presented in frequencies (n) and proportions (%)

Demographic information	Category	Frequency and Proportion n (%)
Age	Less than 20 years	68 (15.9%)
	More than 20 years	361 (84.1%)
Gender	Male	133 (31.0%)
	Female	296 (69.0%)
Health college	Yes	194 (45.2%)
	No	235 (54.8%)
College and department	Faculty of medicine	95 (22.1%)
	College of nursing	37 (8.6%)
	College of applied medical science	29 (6.8%)
	Faculty of administration science	44 (10.3%)
	Faculty of science and literature	37 (8.6%)
	Others	187 (43.6%)
Academic level	Level one	55 (12.8%)
	Second Level	17 (4.0%)
	The third level	72 (16.8%)
	Fourth level	44 (10.3%)
	Level five	51 (11.9%)
	Sixth level	34 (7.9%)
	Seventh level	41 (9.6%)
	Eighth level	27(6.3%)
	Ninth	18 (4.2%)
	Tenth level	17 (4.0%)
	Eleventh level	11 (2.6%)
	Twelfth level	12 (2.8%)
	Thirteenth level	9 (2.1%)
	Fourteenth level	21 (4.9%)
Nationality	Saudi	424 (98.8%)
	Non-Saudi	5 (1.2%)

Table 2 (below) depicts the clinical history of the participants. The findings revealed that 22.1% (n=95) reported a previous diagnosis of ear problems, 25.4% (n=109) reported a previous diagnosis of nose problems, and 22.1% (n=95) reported a previous diagnosis of throat problems. Moreover, 14.2% (n=61) reported having undergone ear, nose, and throat surgery.

**Table 2:** Clinical history of the participants. The clinical history of participants is presented in frequencies (n) and proportions (%)

Clinical history	Categories	Frequency and Proportion n (%)
Previously diagnosed with ear problems	Yes	95 (22.1%)
	No	334 (77.9%)
Previously diagnosed with nose problems	Yes	109 (25.4%)
	No	320 (74.6%)
Previously diagnosed with throat problems	Yes	95 (22.1%)
	No	334 (77.9%)
Previous surgery concerning ear, nose, throat	Yes	61 (14.2%)
	No	368 (87.8%)

Table 3 (below) depicts the knowledge about problems related to the ear, nose, and throat of participants. The vast majority, 61.1% (n=262), of the participants provided correct answers regarding the safety of using cotton swabs for ear cleaning. 61.8% (n=265) were aware that it is recommended to get flu vaccines annually as a preventive measure. More than half (62.2%, (n=267) of the participants provided the correct answers on the importance of using vitamin C to treat and prevent influenza, and 77.6% (n=333) provided the correct definition of vertigo. Vast majority 80.4% (n=345) of the participants were aware that hearing loss can affect their social lives, with more than half (n (n=253) of the participants aware that constant exposure to loud and boisterous noises or sounds may cause hearing loss. 75.0% (n=324) of the participants had the knowledge that some infections of the inner ear and middle ear cause dizziness. About 43.6% (n=187) of the participants provided the correct answer to the question about handling nose bleeding. However, fewer than 50% of the respondents provided the correct answer to the question about the risk of developing obesity and immunodeficiency after tonsillectomy (29.1%, 42.2%). The vast majority (79%; n=339) of the participants were aware that constant misuse of voice causes vocal cord disorders.

**Table 3:** Questions on knowledge about problems related to the ear, nose, and throat. Questions on knowledge about problems related to ENT are presented in frequencies (n) and proportions (%).

Knowledge about problems related to ENT	Categories	Frequency and Proportion n (%)
Cotton swabs are an unsafe way to clean the ears?	Yes	262 (61.1%)
	No	92(21.4%)
	I don’t know	75(17.4%)
Antibiotics are not considered to treat upper respiratory infections (such as colds or coughs).	Yes	151(35.1%)
	No	116(27%)
	I don’t know	162 (37.8%)
It is recommended to get the flu vaccine annually as a preventive measure	Yes	265 (61.8%)
	No	74(17.2%)
	I don’t know	90 (21.0%)
It is recommended for patients with diabetes and high blood pressure to take the seasonal flu vaccine.	Yes	215 (50.1%)
	No	36 (8.4%)
	I don’t know	178(41.5%)
Taking vitamin C treats and prevents influenza	Yes	267(62.2%)
	No	67(15.6%)
	I don’t know	95(22.1%)
Vertigo is another term for dizziness.	Yes	333 (77.6%)
	No	42 (9.8%)
	I don’t know	54(12.6%)
Hearing loss can affect the social life of the individual.	Yes	345(80,4%)
	No	44 (10.3%)
	I don’t know	40 (9.3%)
Constant exposure to loud and annoying noises or sounds may cause hearing loss.	Yes	253(59.0%)
	No	75 (17.4%)
	I don’t know	101 (23.5%)
Some infections of the inner ear and middle ear cause dizziness.	Yes	324 (75.5%)
	No	25(5.8%)
	I don’t know	80 (18.6%)
The correct way to deal with a nosebleed is to put the head back.	Yes	138(32.1%)
	No	187(43.6%)
	I don’t know	104 (24.2%)
The use of nasal congestion drops is considered safe for long-term use	Yes	90 (21.0%)
	No	135 (31.5%)
	I don’t know	204 (47.6%)
Tonsillectomy may cause immunodeficiency	Yes	181 (42.2%)
	No	90 (21.0%)
	I don’t know	158 (36.8%)
Does constant screaming cause vocal cord disorders?	Yes	339 (79%)
	No	20 (4.7%)
	I don’t know	70 (16.3%)

**Figure 1:**
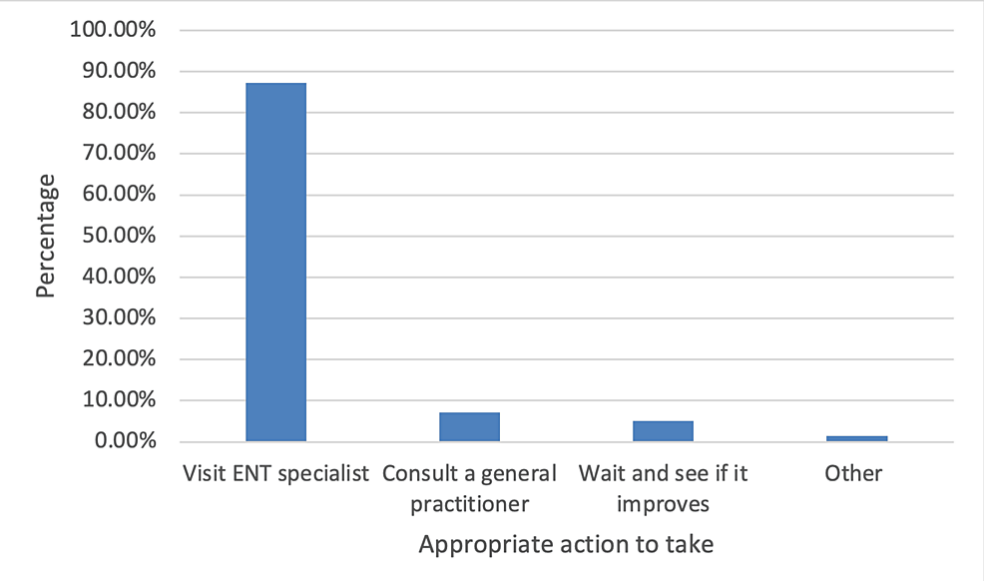
Bar graph depicting responses for the right action that needs to be taken in case of sudden hearing loss or ear when there is sudden hearing loss and earache.

Figure 1 shows the action that respondents should take in case they develop sudden hearing loss or earache. It is evident that most participants would visit an ENT specialist (87.2%), while a significantly smaller proportion would consult a general practitioner (7.2%).

**Figure 2:**
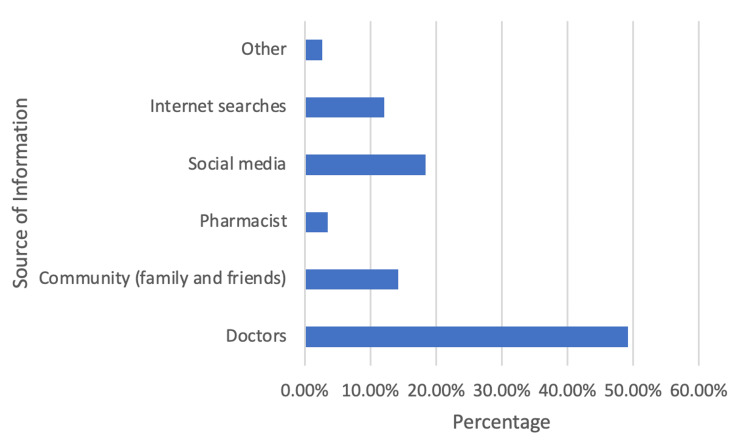
Bar graph showing the sources of information and knowledge about ENT issues.

Figure 2 below shows the participants' sources of information about ENT issues. It is evident that most respondents acquired the information from doctors (49.2%) and social media (18.4%).

Table 4 (below) depicts the relationship between participants' demographics, such as age, gender, health college, college department, nationality, previous clinical history, and level of knowledge about problems related to ENT. The results established a statistically significant association between age, gender, health college, college, and department with p-values<0.005 (0.002*, 0.001*, 0.003, and 0.005*), respectively, and the level of knowledge about problems related to ENT. There was no statistically significant association between nationality, clinical history of the participants, and the level of knowledge about problems related to ENT (p>0.005). The study results show that 37.8% (n=162) had a good knowledge level, while 62.2% (n=267) had poor knowledge about problems related to ENT.

**Table 4:** The association between age, gender, health college, college department, nationality, previous clinical history, and level of knowledge about problems related to ENT. Association between participants’ demographics and knowledge of problems related to ENT * Significant at the p<0.05 level.

	Level of knowledge			
Variables	Category	Poor	Good	p-value
Age	More than 20 years	281 (65.6%)	148 (34.4%)	0.002*
	More than 20 years	275 (64.1%)	154 (35.9%)	
Gender	Male	287 (67.0%)	142 (33.0%)	0.001*
	Female	283 (65.9%)	146 (34.1%)	
Health college	Yes	208 (48.6%)	221 (51.4%)	0.003*
	No	306 (71.4%)	123 (28.6%)	
College and department	Faculty of medicine	121 (28.2%)	308 (71.8%)	0.005*
	College of nursing	160 (37.3%)	296 (62.7%)	
	College of applied medical science	169 (39.5%)	260 (60.5%)	
	Faculty of administration science	288 (67.1%)	141 (32.9%)	
	Faculty of science and literature	284 (66.1%)	145 (33.9%)	
	Others	323 (75.2%)	106 (24.8%)	
Nationality	Saudi	312 (72.8%)	117 (27.2%)	0.341
	Non-Saudi	332 (77.4%)	97 (22.6%)	
Previously diagnosed with ear problems	Yes	256 (59.7%)	173 (40.3%)	0.155
	No	288 (67.1%)	141 (32.9%)	
Previously diagnosed with nose problems	Yes	284 (66.3%)	145 (33.7%)	0.148
	No	279 (65.1%)	150 (34.9%)	
Previously diagnosed with throat problems	Yes	283 (66.0%)	146 (34.0%)	0.371
	No	282 (65.9%)	147 (34.1%)	
Previous surgery concerning ear, nose and throat	Yes	291 (67.9%)	138 (32.1%)	0.537
	No	279 (65.1%)	150 (34.9%)	

**Figure 3:**
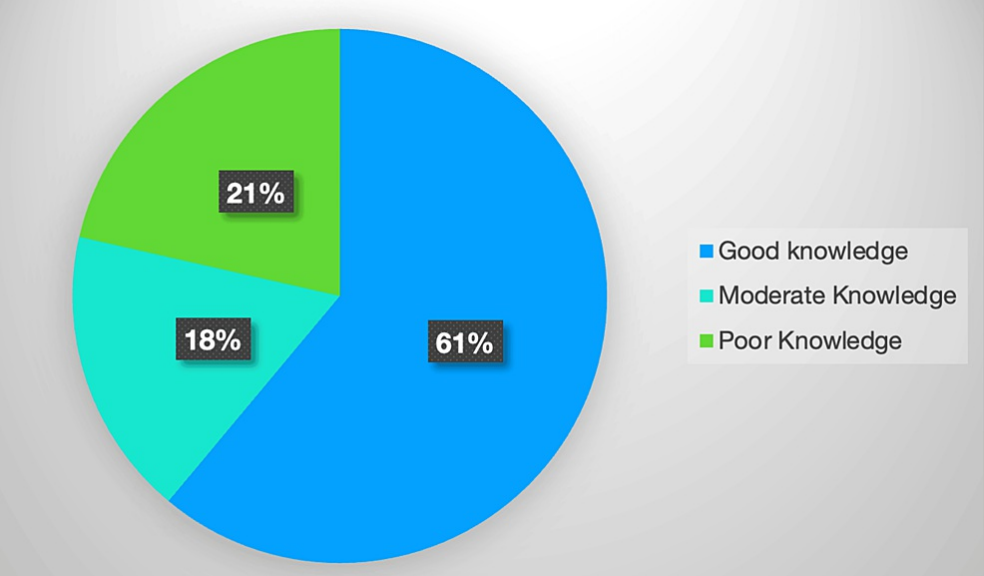
Pie chart showing the knowledge score.

Most of the respondents (61%) showed good knowledge of common ENT problems. About 18% of the respondents had moderate knowledge, while 21% demonstrated poor knowledge, as shown in Figure 3.

## Discussion

The study aims to assess the awareness and practice of Najran University students toward common problems related to the ear, nose, and throat. The sample for the current study primarily consisted of students aged more than 20 years, with a predominance of females. The majority of them were in health colleges, faculties of medicine, and third level, with the majority of them being Saudi nationals.

The findings revealed that only 37.8% (n = 162) of the participants had good knowledge about problems related to ENT. The study showed that participants older than 20 years had better knowledge of common ENT problems than those younger than 20 years. Additionally, female participants showed a higher level of knowledge and awareness. The findings are consistent with the findings of the study conducted by Alassaf et al. (2023), which noted that women had a higher level of knowledge and awareness of problems related to the ears, nose, and throat, considering that locally, they are caretakers of family health issues rather than males in general [11]. Most of the participants showed good knowledge regarding the safety of using cotton swabs for ear cleaning. 61.1% (n = 262) and 61.8% (n = 265) of the participants had good knowledge about vaccinations recommended for the prevention of flu. More than half (62.2%, n = 267) of the participants had good knowledge about the use of vitamin C to treat and prevent influenza, and 77.6% (n = 333) had good knowledge regarding the definition of vertigo. The current findings mirror the findings of a study conducted by Alkholaiwi et al. (2020) in Saudi Arabia, which established good public awareness regarding the potential harms of using cotton swabs for ear cleaning [14]. The findings were also consistent with the findings of a study conducted by Di Berardino et al. (2013) in Milan, Italy, which showed that most of the participants in the study had good knowledge about the damage caused by cotton earbuds [9].

The findings revealed that the majority of the participants had good knowledge that some infections of the inner ear and middle ear cause dizziness and the appropriate measures for handling nose bleeding. The study noted that the majority of the participants, 87.2% (n = 374), had good knowledge with respect to the appropriate action to be taken in case of a sudden hearing problem or earache-going to the hospital-and that the information and knowledge about ENT issues were acquired by the participants from doctors. The findings concur with those of Alshehri et al. (2019), who reported a good level of awareness of going to the hospital as the most appropriate action to be taken in case of a sudden ear, nose, and throat problem [15].

The significant constraint and limitation in this investigation was the employment of a cross-sectional study design, which can only establish relations between factors but not causalities. As this was a survey-based study, recollection bias might be a limitation that also needs further investigation. Also, the study findings cannot be generalized to the entire Saudi Arabian student population, considering that they were conducted at only one institution.

## Conclusions

The study revealed that 37.8% of the participants had good knowledge about problems related to the ears, nose, and throat. The participants older than 20 years had better knowledge of common ENT problems than those younger than 20 years. Female participants showed a higher level of knowledge and awareness of problems related to the ears, nose, and throat. The study noted that the participants in Health College, the faculty of medicine, and the third academic level had good knowledge about problems related to ENT. The study established that going to the hospital was the appropriate action to be taken in case of a sudden ENT pathology. Therefore, we recommend concerted efforts be made among the medical community to increase knowledge about common ENT problems.
